# Computational Study of the H_2_CS and H_2_CO Reactions with CCH: Mechanisms and Kinetic Implications for Interstellar Chemistry

**DOI:** 10.1021/acs.jpca.6c04860

**Published:** 2026-10-12

**Authors:** Silvia Alessandrini, Luigi Crisci, Cristina Puzzarini

**Affiliations:** † 9296 Scuola Superiore Meridionale, Largo San Marcellino 10, 80138 Naples, Italy; ‡ Dipartimento di Chimica “Giacomo Ciamician”, 657489 Alma Mater Studiorum − University of Bologna, Via P. Gobetti 85, I-40129 Bologna, Italy

## Abstract

The high abundance of the ethynyl radical (CCH) in the interstellar medium (ISM) makes its reaction with thioformaldehyde (H_2_CS) potentially important for understanding the chemistry of sulfur-containing molecules and small carbon chains in cold environments. In this work, we present a comprehensive theoretical investigation of this gas-phase reaction through an accurate characterization of the corresponding reactive potential energy surface (PES). To determine reaction rate coefficients and identify the most favorable reaction pathways under the low-temperature and low-pressure conditions of the ISM, kinetic simulations are performed. To assess the reliability of the theoretical approach and elucidate the role of sulfur substitution, the analogous H_2_CO + CCH reaction has been investigated at the same level of theory and directly compared with available experimental kinetic data. Despite the markedly richer and more interconnected PES of the sulfur system, both reactions are overwhelmingly dominated by a barrierless H-abstraction mechanism leading to HCS/HCO and acetylene, thus making the H_2_CS + CCH reaction an inefficient source of the C_3_H_2_S isomers under interstellar conditions. The comparison with the oxygen analogue reveals both common mechanistic features and important differences while validating the adopted computational protocol. Our study provides the first reliable kinetic characterization of the H_2_CS + CCH reaction, delivering rate coefficients suitable for inclusion in astrochemical databases.

## Graphical abstract

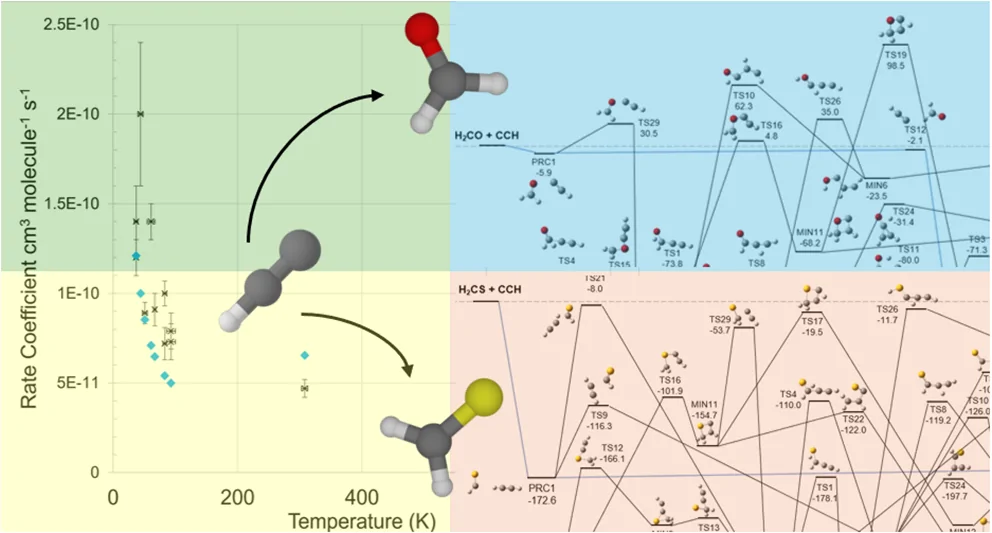

## Introduction

In the past few years, the number of detections of sulfur-bearing species has increased, with several species being observed in the Taurus Molecular Cloud-1 (TMC-1) in 2025 and 2026.^1–3^ Such recent observations have highlighted the presence of three isomers of the C_3_H_2_S family in TMC-1: thiopropynal (HC­(=S)­CCH), cyclopropenethione (c-C_3_H_2_=S), and propadienthione (l-H_2_C=C=C=S). According to ref 3, their abundances are significantly different: l-H_2_C=C=C=S is more abundant than HC­(=S)­CCH, which in turn is more abundant than c-C_3_H_2_=S. Overall, the cumulenic chain is about 15 times more abundant than cyclopropenethione and 1.5 times more abundant than thiopropynal. In view of these discoveries, the investigation of gas-phase reactions involving sulfur-bearing molecules is an important step toward the understanding of the detected molecules, the rationalization of their abundance and, more generally, the development of an exhaustive chemical network of the interstellar medium (ISM).^4–7^

Focusing on the three detected isomers of the C_3_H_2_S family, it is noted that the observed abundance pattern reflects the thermodynamic stability, but the abundance ratios are far from thermodynamic expectations, thus suggesting that kinetic processes are involved, with multiple processes that should be considered. Recently, the three corresponding isomers of the C_3_H_2_O family have been observed in the ISM with a different abundance trend with respect to the thermodynamic expectation, but also to the sulfur counterparts.^3^ In detail, propadienone (l-H_2_C=C=C=O) was observed in TMC-1 in 2026,^3^ while propynal (HC­(=O)­CCH) and cyclopropenone (c-C_3_H_2_=O) were detected, in the same source, in 1988 ^9^ and 2016,^8^ respectively. The relative abundance of these O-bearing isomers differs from the thermodynamic order, with propynal (HC­(=O)­CCH) being more abundant than cyclopropenone (c-C_3_H_2_=O) and propadienone (l-H_2_C=C=C=O), despite being second in the stability order.

The chemistry of the oxygen-bearing isomers is somewhat well constrained in the literature thanks to a detailed work reported in 2016 by Loison et al.,^8^ who pointed out that the formation of these species is dominated by gas-phase chemistry.^3,8^ The pathways for the formation of C_3_H_2_O isomers are summarized in Figure 1, where solid lines indicate the well-established gas-phase pathways, while dashed lines refer to pathways that play a minor role. Dotted lines are instead processes that have never been studied in the literature, which are however expected to be relevant for the network. It is evident from Figure 1 that the main routes for forming the O- and S-bearing compounds are different, with those involving sulfur-containing species being nearly uninvestigated.

**Figure 1:**
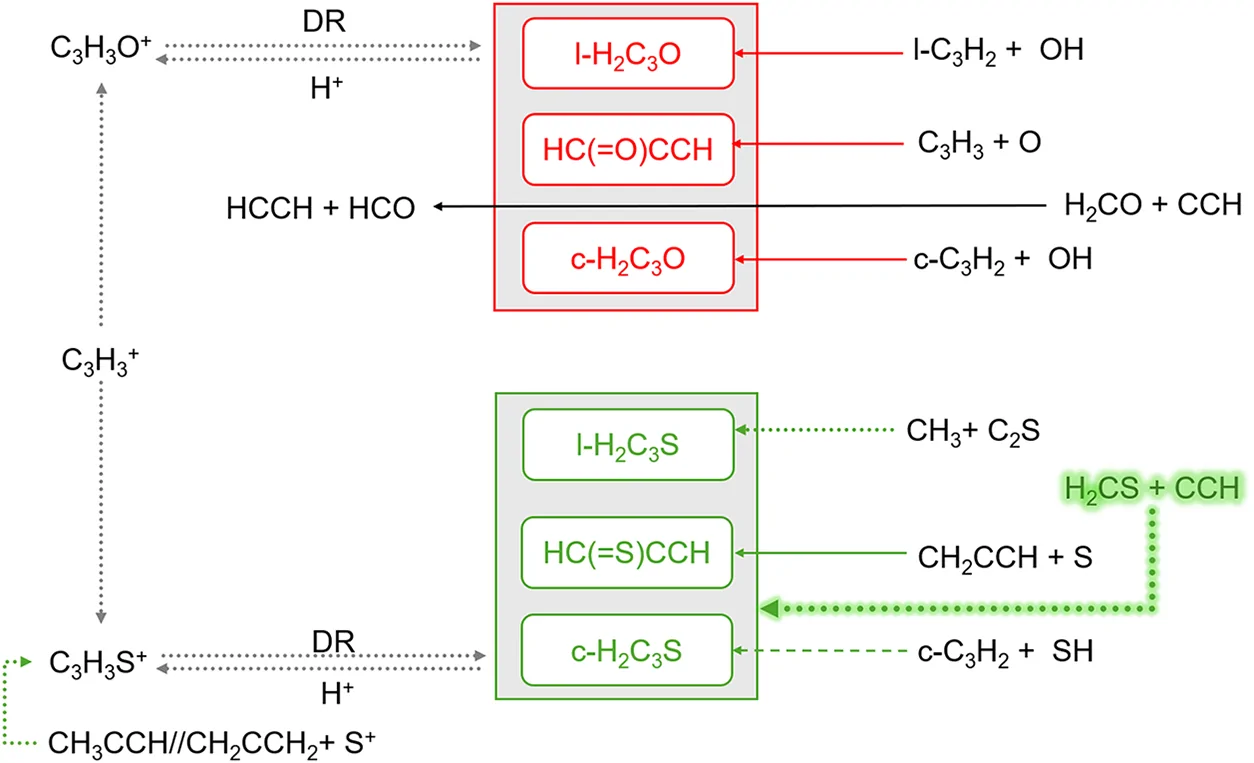
Gas-phase formation networks of the C_3_H_2_O and C_3_H_2_S isomers as illustrated in the literature (see refs 1,3,8 and references therein). Solid lines indicate well-established gas-phase pathways, while dashed lines are minor pathways. Dotted lines denote those processes that have never been studied in the literature, which are however expected to be relevant for the network. The coproducts are implicit.

In this context, identifying and characterizing gas-phase reactions leading to the formation of the C_3_H_2_O/C_3_H_2_S isomers becomes essential when trying to understand the relative abundances within a family and between the members of different families. The present work aims at investigating the role played by the gas-phase H_2_CS + CCH reaction in the sulfur network depicted in Figure 1. Both reactants are abundant in TMC-1 ^10,11^ and the reaction mechanism investigated could provide additional insights into the rationalization of the relative abundances of the C_3_H_2_S isomers. To support the comparison with the oxygen counterparts, this work also presents the study of the H_2_CO + CCH reaction, which was theoretically studied in 2005 by Dong et al.^12^ and recently experimentally investigated in ref 13. According to the literature, the latter reaction predominantly leads to H-abstraction, thus forming HCCH + HCO and, consequently, having no relevant role in the interstellar formation of the C_3_H_2_O isomers. However, our recent investigation of the H_2_CO + CN and H_2_CS + CN reactions pointed out that, while the former is entirely dominated by H abstraction, the latter predominantly leads to addition, thus forming HC­(S)­CN.^14^

In the following, the methodology employed to study both reactions is introduced, then the results will be illustrated focusing on the reactive potential energy surfaces (PESs) first and on the kinetic outcomes then. Lastly, concluding remarks are presented.

## Methodology

The strategy employed in the present work to explore the reactive PES involves the following steps:^15^

1. A preliminary study of the reactive PES is performed using the B3LYP functional^16,17^ in conjunction with a partially augmented double-ζ basis set (jun-cc-pVDZ).^18,19^ These calculations also include the D3BJ dispersion correction.^20,21^ In the following, this level of theory is shortly denoted B3/junDZ. Whenever a transition state (TS) is located, Intrinsic Reaction Coordinate (IRC) calculations^22^ are carried out to move forward in the PES exploration. The Hessian is computed at all stationary points to check their nature.
2. Once the PES exploration at the B3/junDZ level is completed, the stationary points’ structures are reoptimized using the rev-DSDPBEP86 functional^23^ to improve their accuracy. For this step, a partially augmented triple-ζ basis set is employed (jun-cc-pV­(T+d)­Z set,^18,19,24^ where the “+d” refers to sulfur and denotes the d-augmented variant). Overall, this level of theory, which incorporates the D3BJ correction, is hereafter denoted as revDSD/junTZ. At this level of theory, the Hessian is also recomputed, thus providing the harmonic zero-point energy (ZPE) correction. Furthermore, if the TS geometry at this level of theory drastically changes from that from the B3/junDZ calculations, IRC calculations are also recomputed.
3. On top of the revDSD/junTZ geometries, the electronic energy is further improved using the junChS composite scheme^14,25^ (*vide infra*).

The starting point of the junChS approach is the energy calculation using the CCSD­(T) method (the acronym indicating the coupled cluster method with single and double excitations and a perturbative treatment of connected triples^26^) together with the jun-cc-pV­(T+d)­Z basis set (shortly denotes as CC/junTZ), within the frozen-core (fc) approximation. To this term, two corrections are added in order to account for the extrapolation to the complete basis set (CBS) limit (Δ*E*_MP2_^CBS^) and the core-valence (CV) correlation effects (Δ*E*_MP2_^CV^). These corrections are computed using second-order Møller–Plesset perturbation theory (MP2)^27^ as follows

1 $$\Delta E_{\mathrm{MP}2}^{\mathrm{CBS}}=\frac{4^{3}E_{\mathrm{fc}-\mathrm{MP}2}^{\mathrm{junQZ}}-3^{3}E_{\mathrm{fc}-\mathrm{MP}2}^{\mathrm{junTZ}}}{4^{3}-3^{3}}-E_{\mathrm{fc}-\mathrm{MP}2}^{\mathrm{junTZ}}$$

and

2 $$\Delta E_{\mathrm{MP}2}^{\mathrm{CV}}=E_{\mathrm{ae}-\mathrm{MP}2/\mathrm{cc}-\mathrm{pwCVTZ}}-E_{\mathrm{fc}-\mathrm{MP}2/\mathrm{cc}-\mathrm{pwCVTZ}}$$

Above, the CBS term (eq 1) is determined by applying the *n* ^–3^ extrapolation formula^28^ in conjunction with the jun-cc-pV­(T+d)­Z (*n* = 3) and jun-cc-pV­(Q+d)­Z (*n* = 4) basis sets, indicated as junTZ and junQZ, respectively. The CV contribution is obtained as the difference between energy calculations correlating all electrons (ae) and within the fc approximation in the same basis set (the cc-pwCVTZ basis set^29^). By combining the contributions presented above, the junChS energy is obtained as

3 $$E\left(\mathrm{junChS}\right)=E\left(\mathrm{CC}/\mathrm{junTZ}\right)+\Delta E_{\mathrm{MP}2}^{\mathrm{CBS}}+\Delta E_{\mathrm{MP}2}^{\mathrm{CV}}$$

Once the reactive PES is characterized, kinetic simulations are performed. The theoretical approach for deriving rate constants for a multiwell, multiarrangement reaction involves treating each elementary step separately. Transition state theory (TST)^30^ is employed to calculate the rate constants for the elementary steps involving a TS. Specifically, the rate coefficient for a unimolecular elementary step is determined using the Rice-Ramsperger-Kassel-Marcus (RRKM) theory.^31,32^ For modeling the barrierless entrance and exit channels, the phase space theory (PST) has been used.^33,34^

In the PST framework, the isotropic long-range interaction between the reactants is modeled by an effective potential

4 $$V_{n}\left(R\right)=-\frac{C_{n}}{R^{n}},\mathrm{with}\;\,V\left(\infty \right)=0$$

where *n* = 4, 5, or 6. The term adopted in the PST calculation is that resulting from the smallest root-mean-square deviation of the fit (RMSD) of the reference data. The values obtained will be discussed in the results section, together with the derivation of the rate coefficients. The interaction energies entering the fit were computed at the revDSD/junTZ and junChS-F12 ^35^ levels of theory. The latter scheme is similar to the above-mentioned junChS approach, but involves the corresponding explicitly correlated (F12) methods (i.e., CCSD­(T)-F12a^36^ and MP2-F12 ^37^). For each barrierless channel, the appropriate scan of the reaction was selected following the strategy described in ref 38. The specific distances employed for each channel are collected in the Supporting Information (SI), which also reports the comparison between different fitting procedures. Readers interested in the full validation of the employed strategy are referred to ref 38.

Finally, for each pathway, the global rate constant is derived from the resolution of the master equation, which integrates the PST and RRKM rate coefficients. Kinetic simulations have been performed using the master equation system solver (MESS) program.^39^ For the H_2_CO + CCH reaction, the simulations have been carried out in the range *T* = 10–603 K in order to compare the computed results with experimental data, while for H_2_CS + CCH, the simulations have been limited to the range between 30 and 300 K.

For both reactions, the temperature dependence of the rate constants has been modeled using the three-parameter Arrhenius-Kooij formula:^40^

5 $$k\left(T\right)=\alpha {\left(\frac{T}{300}\right)}^{\beta }\exp\left(-\frac{\gamma }{T}\right)$$

where α, β, and γ are the fitting parameters: α represents the pre-exponential factor at the reference temperature of 300 K, β accounts for its temperature dependence, providing a correction for nonstandard reaction dynamics, and γ is related to the effective activation energy, reflecting the temperature effects on the rate constant.

The F12 calculations were carried out using Molpro 2025.3,^41–43^ while the DFT and junChS computations were performed with the Gaussian16 suite of program (C02 revision).^44^

## Results and Discussion

### Reactive Potential Energy Surfaces

The discussion will focus on the description of the H_2_CS + CCH PES first and then move to the comparison with its oxygen counterpart. Following that, we will delve into the kinetic results and their astrochemical implications.

#### The H_2_CS + CCH Reaction

The reactive PES for the H_2_CS + CCH reaction is shown in Figure 2, which reports the relative energies (with respect to the reactants’ asymptote) at the junChS level and corrected for the harmonic revDSD/junTZ ZPE contribution. In Table 1, these energies are compared with those entirely obtained at the revDSD/junTZ level of theory.

**Figure 2:**
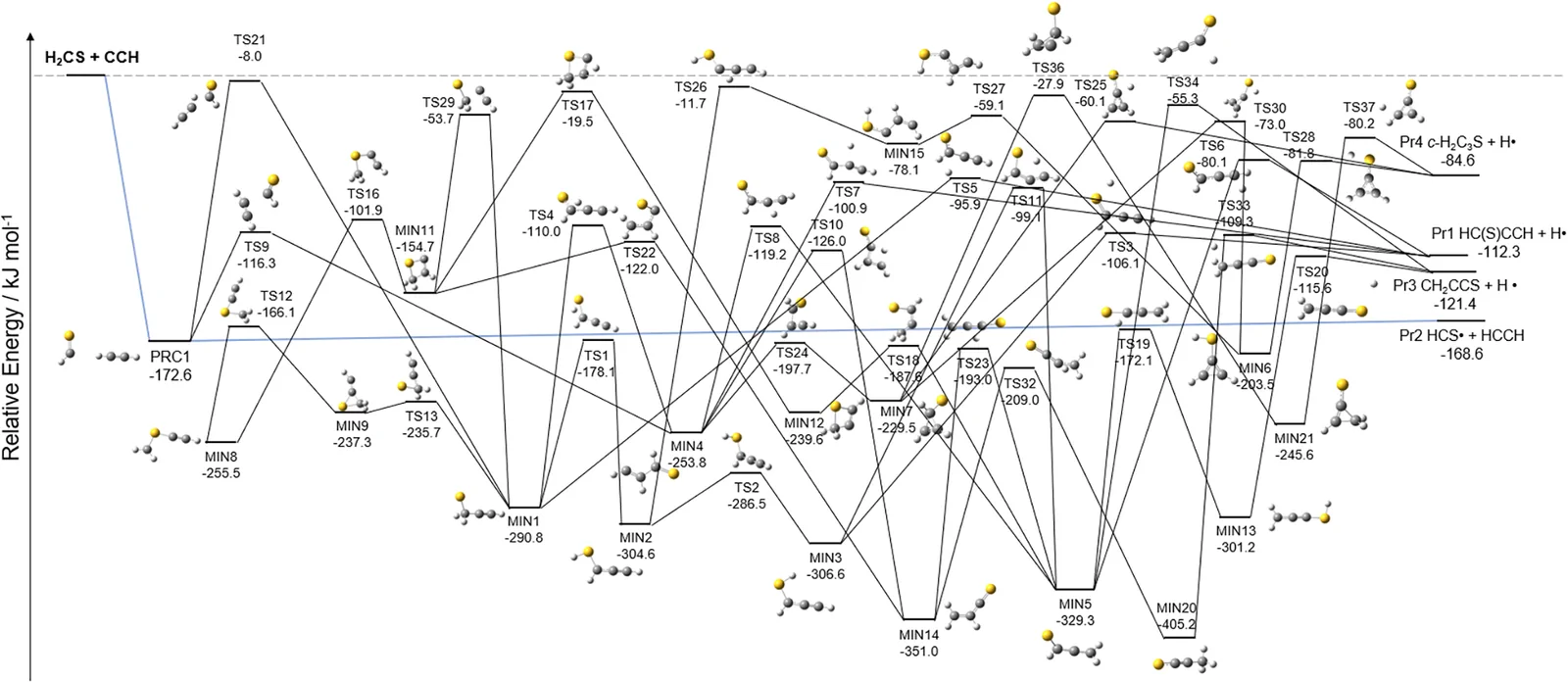
H_2_CS + CCH PES: The junChS relative energies corrected for the harmonic revDSD/junTZ ZPE contribution are reported. The main pathway is highlighted in blue.

**Table 1:** Relative Energies (kJ·mol^–1^)­^a^ ^,^ ^b^ of the Stationary Points on the H_2_CS + CCH PES

	revDSD/junTZ	junChS		revDSD/junTZ	junChS		revDSD/junTZ	junChS
**PRC1**	– 193.9	–172.6	**TS2**	–313.3	–286.5	**TS22**	–131.8	–117.4
**MIN1**	–328.5	–290.8	**TS3**	–119.0	–106.1	**TS23**	–223.6	–193.0
**MIN2**	–332.1	–304.2	**TS4**	–124.5	–105.9	**TS24**	–206.6	–190.3
**MIN3**	–334.3	–306.6	**TS5**	–107.3	–95.9	**TS25**	–88.8	–60.1
**MIN4**	–269.2	–253.8	**TS6**	–105.4	–80.1	**TS26**	–30.8	–11.7
**MIN5**	–362.2	–329.3	**TS7**	–87.0	–100.9	**TS27**	–72.2	–59.1
**MIN6**	–218.84	–203.5	**TS8**	–124.2	–119.2	**TS28**	–113.6	–81.8
**MIN7**	–264.4	–229.5	**TS9**	–141.8	–116.3	**TS29**	–82.6	–53.7
**MIN8**	–288.1	–255.5	**TS10**	–139.0	–126.0	**TS30**	–101.4	–73.0
**MIN9**	–265.6	–237.3	**TS11**	–135.0	–99.1	**TS32**	–200.7	–209.0
**MIN11**	–182.4	–154.7	**TS12**	–185.5	–166.1	**TS33**	–125.3	–109.3
**MIN12**	–264.82	–239.6	**TS13**	–264.0	–235.7	**TS34**	–53.9	–55.3
**MIN13**	–331.9	–301.2	**TS16**	–107.5	–101.9	**TS37**	–100.5	–80.2
**MIN14**	–381.7	–351.0	**TS17**	–42.0	–19.5	**TS36**	–46.2	–27.2
**MIN15**	–99.7	–78.1	**TS18**	–193.1	–187.6	**Pr4**	–118.2	–84.6
**MIN20**	–393.4	–405.2	**TS19**	–190.9	–172.1	**Pr3**	–153.6	–121.4
**MIN21**	–271.6	–245.6	**TS20**	–125.2	–115.6	**Pr2**	–193.3	–168.6
**TS1**	–199.8	–178.1	**TS21**	–30.6	–8.0	**Pr1**	–141.9	–112.3

a The ZPE-corrected absolute energies of the reactants are, at the revDSD/junTZ level, −437.07918 *E*_h_ for H_2_CS and −76.46258 *E*_h_ for CCH; using the junChS scheme, −437.30846 *E*_h_ for H_2_CS and −76.58286 *E*_h_ for CCH.

b Harmonic ZPE corrections are calculated at the revDSD/junTZ level of theory. For labeling, see Figure 2.

From the inspection of Figure 2, it is noted that all C_3_H_2_S isomers are formed together with the H·atom as a coproduct, and their stability order follows the trend already observed in the literature: the cumulenic chain l-H_2_C=C=C=S (Pr3) is the most stable form, with thiopropynal (Pr1) and cyclopropenethione (Pr4) being located 9.1 and 36.8 kJ mol^–1^ higher in energy, respectively. This is in agreement with ref 1, where the Pr1 and Pr4 are found to lie 10.5 and 38.2 kJ mol^–1^ above Pr3, respectively. This agreement was somewhat expected considering that the energies there reported were computed at the CCSD­(T)/CBS level of theory on top of M06–2X-D3/def2-TZVPP optimized geometries. In Figure 2, it is apparent that the most stable product is HCS + HCCH (Pr2), which lies 47.2 kJ mol^–1^ below Pr3.

An important outcome of the exploration of the H_2_CS + CCH PES is that all stationary points are located below the reactants’ energy, resulting in a highly interconnected reaction network. The investigation of the reactive entrance channel reveals a barrierless process that directly evolves into a prereactive complex (PRC1) where the H-abstraction has already occurred. PRC1, lying 172.6 kJ mol^–1^ below the reactants, directly connects to the Pr2 product (−168.6 kJ mol^–1^) through a barrierless channel characterized by an energy difference as small as 4 kJ mol^–1^. From PRC1, the reaction can also proceed forward to MIN1 by overcoming a barrier of about 165 kJ mol^–1^ (ruled by TS21), or to MIN4 through a barrier of ∼56 kJ mol^–1^ due to TS9. An intricate pattern is accessed from these minima, and the main processes observed are H-migrations between the heavy atoms (carbon and sulfur) and cyclization processes involving C–C and C–S bond formation. The latter processes provide the only possible pathway for atom rearrangements for submerged steps. As an example, MIN9 is a cyclic intermediate that offers the possibility to transfer a −CCH group from the sulfur atom of H_2_CS to the carbon atom of the same molecule. Overall, the H_2_CS + CCH PES has a large number of energetically accessible addition, migration, and cyclization pathways. However, even if these are energetically accessible, their corresponding kinetic relevance needs to be addressed (see section Kinetic Analysis and Astrochemical Implications).

#### The H_2_CO + CCH Reaction and S/O Comparison

The H_2_CS + CCH PES can be compared with the H_2_CO + CCH one, which – as mentioned in the Introduction – has already been investigated computationally^12^ and experimentally.^13^ Reconsidering the O-bearing reactive PES aims to address the differences with respect to the H_2_CS + CCH one in terms of entrance channels and energetic behavior, but also to estimate the error affecting our kinetic simulations and, in particular, the rate coefficients (*vide infra*).

The H_2_CO + CCH PES is depicted in Figure 3, where the junChS relative energies, corrected for the harmonic revDSD/junTZ ZPE contributions, are reported. The comparison between the revDSD/junTZ and junChS energies is provided in Table S10 of the SI.

**Figure 3:**
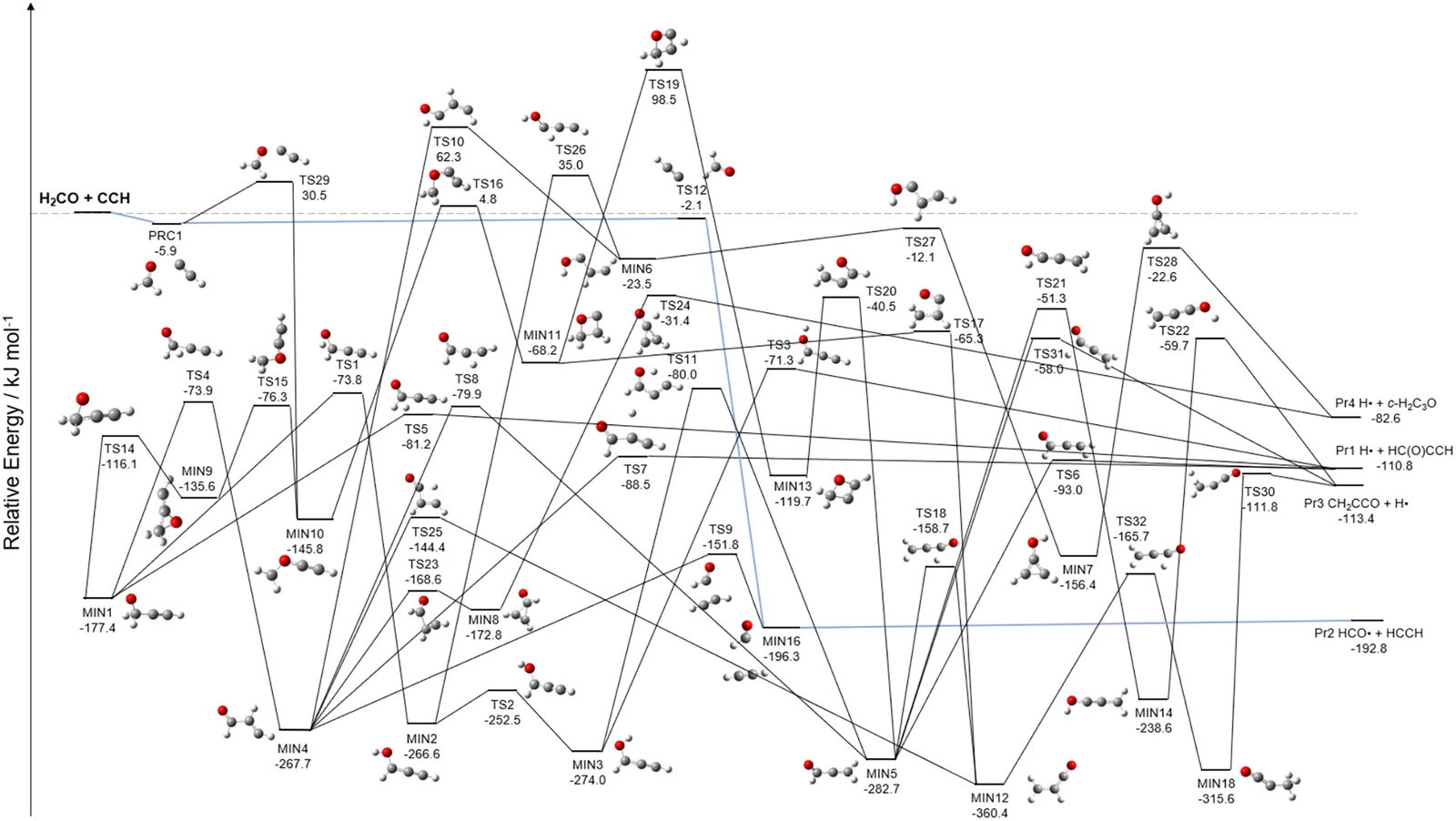
H_2_CO + CCH PES: The junChS relative energies corrected for the harmonic revDSD/junTZ ZPE contribution are reported. The main pathway is highlighted in blue.

In agreement with the literature,^12,13^ the reactants’ approach is barrierless and leads to the formation of the prereactive complex (PRC1), which can evolve into a weakly bound system between the HCO radical and HCCH (MIN16) by overcoming a submerged barrier of 3.8 kJ mol^–1^ (ruled by TS12 located at −2.1 kJ mol^–1^). MIN16 proceeds without any barrier into Pr2 (HCO + HCCH), which is the most exothermic product on the PES (−192.6 kJ mol^–1^). This pathway closely resembles the one of the H_2_CS + CCH system, the main difference being the lack of an intermediate in the entrance channel in the case of H_2_CS + CCH.

The O-containing PES reveals the presence of an emerged TS (TS29 at +30.5 kJ mol^–1^) for the formation of a C–O bond between the O atom of H_2_CO and the terminal C atom of CCH. A similar mechanism was not observed in the sulfur counterpart, even if several attempts and different starting geometries were considered. Indeed, in the case of H_2_CS + CCH, the optimization either evolved into MIN1, MIN8, or TS29, all being characterized by the formation of a C–C bond. The IRC calculation for TS29 of the sulfur PES, at the revDSD/junTZ level, pointed out that the minima connected by this TS are MIN11 and MIN1 (see Figure 2) and that the TS is not directly connected to the reactants.

Moving back to the H_2_CO + CCH PES, the formation of four-membered cycles is inhibited by emerged barriers as those ruled by TS16, which lies at +4.8 kJ mol^–1^, and TS19 located 98.5 kJ mol^–1^ above the reactants. As already noted for the H_2_CS + CN and H_2_CO + CN reactions,^14^ when moving from sulfur to oxygen, several TSs become emerged. To give an example, TS19 of the oxygen system is similar to TS17 of the sulfur counterpart, but the difference in energy is striking. The former is located at nearly 100 kJ mol^–1^ above the reactants, while the latter lies at −19.5 kJ mol^–1^. The global minimum on the PES changes in moving from the oxygen-bearing to the sulfur-containing PES: the vinyl-like structure (MIN12) is the global minimum (−360.4 kJ mol^–1^) for the former, while the cumulenic chain ending with the CH_3_ group (MIN20) is the global minimum for the latter (−405.2 kJ mol^–1^).

Overall, the stability order of the products is similar between the two PESs, but some differences have been observed with respect to the literature data.^8,45^ According to them, the most stable C_3_H_2_O isomer is propadienone (l-H_2_C=C=C=O), this result being in agreement with the outcomes of the present work. However, our calculations point out that the propynal form (HC­(=O)­CCH) lies only 2.6 kJ mol^–1^ above l-H_2_C=C=C=O, which is quite in contrast with the value of 22.7 kJ mol^–1^ reported in ref 45 but in very good agreement with a previous paper that suggested a difference of only 2.5 kJ mol^–1^.^46^

To clarify this discrepancy, we have computed the electronic energy of propadienone and propynal using the HEAT-like composite scheme^47,48^ on top of the CCSD­(T)/CBS+CV geometries, determined in ref 49. While details on the CCSD­(T)/CBS+CV scheme can be found in the latter reference, here we mention that it is entirely based on the CCSD­(T) method and accounts for the CBS and CV corrections. Concerning the HEAT-like protocol, the reader is referred to refs 47,48,50,51 . Here, we note that this is an approach that, starting from the CCSD­(T)/CBS+CV level, includes up to quadruple excitations in the cluster expansion. In the present case, quadruples have been incorporated in a noniterative manner (exploiting the CCSDT­(Q) method^52,53^ ). Overall, the HEAT-like scheme is expected to provide relative energies with a 1 kJ mol^–1^ accuracy. Back to the l-H_2_C=C=C=O and HC­(=O)­CCH energy difference, the HEAT-like result, including anharmonic ZPE correction at the revDSD/junTZ level, is 3.5 kJ mol^–1^. The high accuracy of the HEAT-like model together with that of the W2–F12 ^54^ scheme employed in ref 46 confirm that the junChS approach is a powerful tool to determine accurate energetics, as required in the investigation of gas-phase reactions under interstellar conditions. Finally, junChS predicts the cyclic isomer to be 30 kJ mol^–1^ less stable than the cumulenic chain, this result being somewhat in agreement with that of ref 45 and in perfect agreement with the value of 29.2 kJ mol^–1^ reported by Karton and Talbi.^46^

Lastly, one can compare the performance of the two levels of theory considered in this work, revDSD/junTZ and junChS. It is noted that the average difference, when moving from revDSD/junTZ energies to junChS, is +11.7 kJ mol^–1^ for the H_2_CO + CCH PES and around +19.1 kJ mol^–1^ for H_2_CS + CCH. The largest deviation (+23 kJ mol^–1^) is observed for MIN18 in the case of the O-containing PES, while the maximum deviation is +37.7 kJ mol^–1^ for MIN1 of the sulfur counterpart. These differences were expected according to previous works.^14,55,56^ Finally, we note that the relative energies computed for the H_2_CO + CCH system in this work are in good agreement with those reported in the literature.^12,13^

### Kinetic Analysis and Astrochemical Implications

As mentioned in the Methodology section, the characterization of both reactive PESs was followed by the kinetic simulations for the derivation of the global rate coefficients, with the experimental data for the H_2_CO + CCH system providing the means for estimating the uncertainties affecting our results. As already described, the barrierless entrance and exit channels have been modeled using PST which translates into deriving accurate *C*_*n*_ parameters. To this aim, we have employed the strategy described in ref 38. In detail, starting from the complete revDSD/junTZ curves, five “core” points have been selected for junChS-F12 energy evaluations and then fitted, according to eq 4, to different exponents. In most of the cases, the best fit was obtained using the *n* = 4 exponent, with the corresponding coefficients being:

- For the entrance channel of the H_2_CO + CCH system, the coordinate is the R = O­(H_2_CO)···C­(HCCH) distance: *C*_4_ = 422.2 Å^4^ kJ mol^–1^.
- For the exit channel leading to the formation of HCO + HCCH (Pr2), the coordinate is the R = C­(HCO)···C­(CCH) distance: *C*_4_ = 753.0 Å^4^ kJ mol^–1^.
- For the entrance channel of H_2_CS + CCH, leading to PRC1, the coordinate is the R = C­(H_2_CS)···C­(CCH) distance: *C*_5_ = 1706.3 Å^5^ kJ mol^–1^.
- For the exit channel leading to HCS + HCCH (Pr2), the coordinate is the R = H­(HCCH)···C­(HCS) distance: *C*_4_ = 350.12 Å^4^ kJ mol^–1^.

A full account on the *C*_*n*_ parameters obtained using different exponents and different numbers of selected points in the fitting procedure is detailed in the SI (Tables S2–S9 and Figures S1–S4). However, it should be mentioned that the coefficients change only up to 0.5% with respect to those reported above, the only exception being noted for the entrance channel of the H_2_CS + CCH reaction. In this last case, the energy trend of the revDSD/junTZ and junChS-F12 scans are different. The revDSD/junTZ coefficients were considered because they lead to the smallest root-mean-square deviation.

Figure 4 shows the comparison between computed and experimental rate coefficients for the H_2_CO + CCH → HCO + HCCH reaction, i.e., the pathway highlighted in blue in Figure 3. It can be noticed that the agreement between computed and experimental results is quite good at low temperatures, with an average deviation of about 17%. However, the discrepancies increase significantly at temperatures above 300 K, with a disagreement of about 160% at *T* = 603 K. While a worsening of the error is expected at high temperatures due to the PST modeling,^14^ such a large disagreement was unexpected and somewhat puzzling. The comparison of the computed vibrational frequencies with experimental data (see Table S11 of the SI)^57–60^ shows that most of the vibrational frequencies used for the computation of the partition functions of products and reactants are correct, with errors ranging between 3 to 5%, except for the bending mode of the CCH radical. This is wrongly predicted: it is computed at 569 cm^–1^ and its experimental value is 371.60 cm^–1^,^61^ thus showing a discrepancy of 53%. This is due to the vibronic coupling between the electronic ground state of CCH and its first excited state, a well-known issue in the literature.^62^ Correcting this frequency (that is, replacing the computed value with the experimental one) in our kinetic simulations lowers *k*(603 *K*) from 1.55 × 10^–10^ to 9.69 × 10^–11^ cm^3^ molecule^–1^ s^–1^, thus decreasing the discrepancy from ∼160% to about 65%. This value is by far more reasonable and indeed ascribable to the use of PST for modeling the entrance and exit channels. At the same time, this empirical correction allows us to completely recover the error observed at 308 K, the discrepancy on the rate coefficient reducing from 39 to 9%.

**Figure 4:**
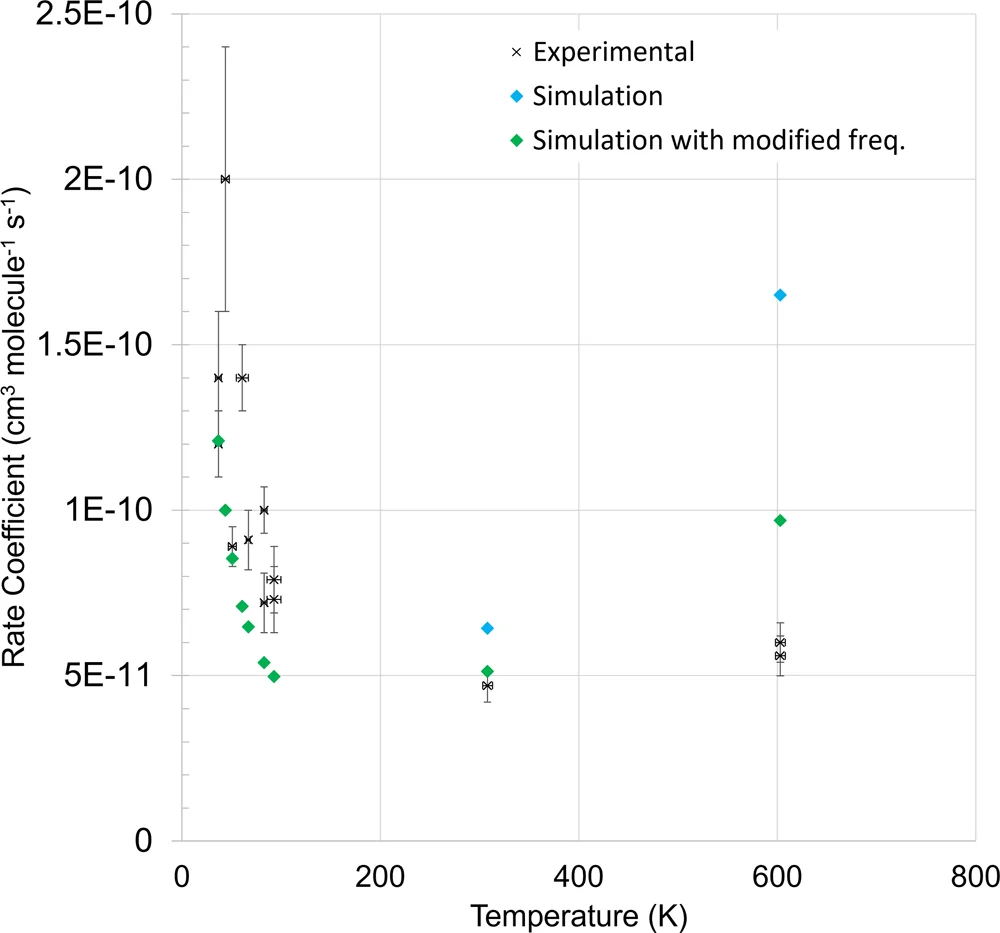
Comparison between computed (diamonds) and experimental^13^ (black cross) rate coefficients for the H_2_CO + CCH → HCO + HCCH reaction. In detail, the light-blue diamonds are the rate coefficients derived using computed data, while the green diamonds are those obtained by replacing the CCH bending frequency with the experimental value (for details, see text).

While the use of the experimental vibrational frequency has a marginal effect on the ZPE correction, it becomes crucial in the derivation of the vibrational partition function of the CCH radical. In fact, lowering the bending frequency increases the vibrational partition function of CCH, thus making excited bending levels more accessible. In PST, the bimolecular rate coefficient strongly depends on the reactants’ partition functions. Therefore, increasing the CCH partition function results in a lower rate coefficient. This effect becomes more pronounced with rising temperatures, as higher bending levels become populated, while it remains nearly negligible at low temperatures. Thus, using the experimental CCH bending frequency provides a realistic statistical description of the reactant states entering the ME treatment, without the need of resorting to multireference methodologies for computing an accurate value (which is the case whenever there is a lack of experimental data). In passing we note that a refined computational treatment of the CCH bending mode was carried out in ref 62 and provided a value of 361 cm^–1^. Finally, it should be mentioned that the experimental rate coefficient obtained at *T* = 44 K for H_2_CO + CCH → HCO + HCCH was excluded from the current statistical estimation because it is an evident outlier on the basis of the trend of the experimental rate coefficients versus temperature. Indeed, the *T* = 44 K value is higher than the previous (at lower temperature) and the following one (at higher temperature), even if error bars are accounted for.

Based on the comparison discussed above, we employed the experimental frequency value of the CCH bending also in the kinetic simulations for the H_2_CS + CCH reaction. This allows us to estimate the error affecting our computed data to be about 15–20%, on average, in the range of *T* = 30–300 K. The kinetic simulations for the H_2_CS + CCH reaction include the pathway highlighted in blue in Figure 2 (leading to HCS + HCCH) as well as all the other accessible channels originating from the path PRC1 → TS9/TS21. The formation of HCS + HCCH is predominant at all the temperatures considered, the trend of the corresponding rate coefficient being shown in Figure 5.

**Figure 5:**
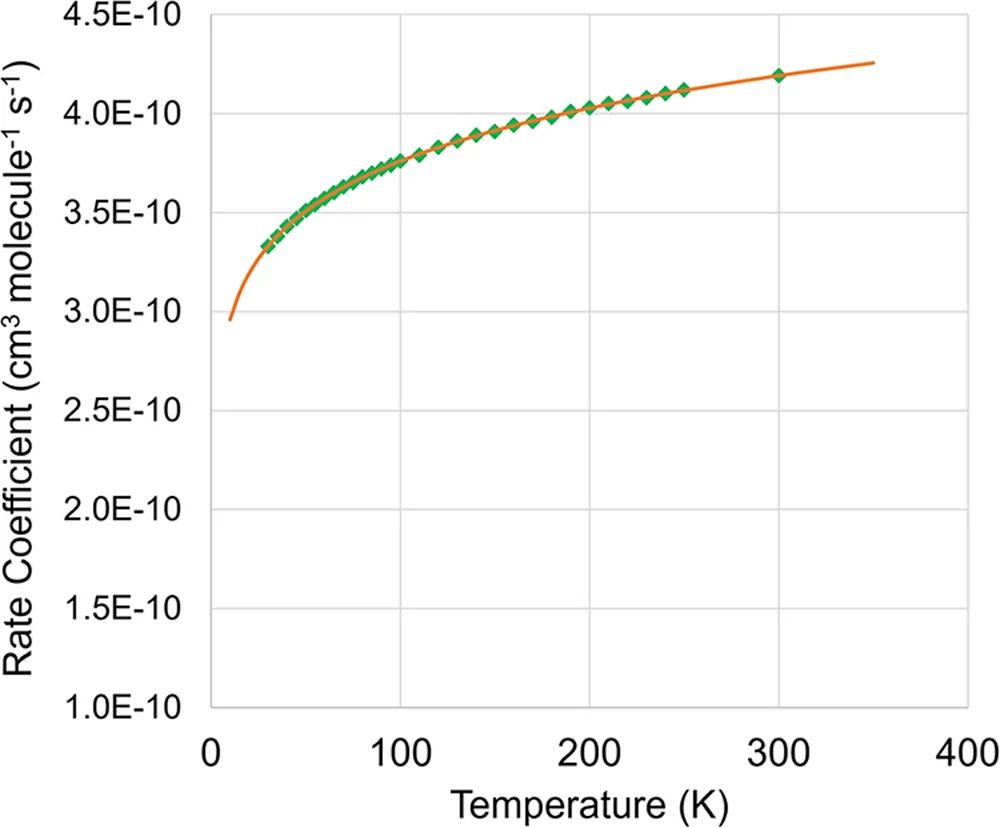
Kinetic simulations of the H_2_CS + CCH → HCS + HCCH reaction: Rate constants (green diamonds) are computed in the 30–300 K temperature range, at a pressure of 1 × 10^–12^ atm, and plotted with the Arrhenius-Kooij expression (orange line).

From the inspection of Figure 5, it is noted that the reaction proceeds with a rate of about 3.6–4.2 × 10^–10^ cm^3^ molecule^–1^ s^–1^ in the entire range considered, thus showing a small temperature dependence. This can be explained by the fact that PRC1 is formed in the entrance barrier and it can easily evolve toward the formation of Pr2 without any barrier. Overall, even if the rate coefficient slowly decreases by decreasing temperature, we expect the reaction to be fast even at 10 K, with an expected rate coefficient of 2.9 × 10^–10^ cm^3^ molecule^–1^ s^–1^. This is due to the fact that, whenever no barriers are present, the rate proceeds at the collisional rate. Figure 5 also shows the curve resulting from the Arrhenius-Kooij plot. The corresponding parameters are collected in Table 2. As mentioned in the Methodology section, for the H_2_CS + CCH reaction, the 30–300 K range has been employed, while for H_2_CO + CCH the temperature interval considered is 10–100 K. Lastly, we note that both reactions are pressure independent, as demonstrated by Figure S5 of the SI.

**Table 2:** Arrhenius-Kooij Parameters for the H_2_CS + CCH and H_2_CO + CCH Reactions^a^

*T* range	product	α/cm^3^ molecule^–1^ s^–1^	β	γ/K	rms^b^
30–300	Pr2, HCS + HCCH	4.19 ± 0.001 × 10^–10^	0.090 ± 0.001	0.15 ± 0.06	2.6 × 10^–13^
10–100	Pr2, HCO + HCCH	1.17 ± 0.06 × 10^–11^	–1.22 ± 0.03	6.1 ± 0.62	3.07 × 10^–12^

a The fit employed the ZPE-corrected junChS relative energies (Table 1).

b rms stands for root-mean-square deviation of the fit.

The last comment concerns two transition states of the H_2_CS + CCH reaction lying close to the reactant asymptote: TS21 and TS26, which are located at −8.0 and −11.7 kJ mol^–1^, respectively. The uncertainty associated with our methodology is expected to be within 5 kJ mol^–1^. Therefore, we expect that these transition states remain submerged with respect to the reactants even if higher levels of theory are employed. However, even if these stationary points had been emerged with respect to the reactants, the main kinetic conclusions would have been not affected because TS21 and TS26 belong to minor rearrangement pathways, whereas the barrierless nature of the dominant H-abstraction remains unchanged.

The comparison carried out in the present work demonstrates that replacing O with S qualitatively and quantitatively modifies the topology and connectivity of the reactive PES. Several rearrangement pathways, inaccessible because of emerged barriers in the oxygen-containing system, become submerged in the sulfur counterpart. Nevertheless, both reactions exhibit the same dominant kinetic outcome because the barrierless H-abstraction channel dominates over the alternative pathways. At temperatures below 10 K, the H_2_CS + CCH reaction is predicted to be slower than H_2_CO + CCH, albeit analogously leading to the H-abstraction product. Finally, it should be pointed out that the H_2_CS + CCH reaction network derived in this work allows for improving the current understanding of sulfur chemistry in cold interstellar environments and provides a benchmark for future laboratory studies.

From an astrochemical viewpoint, the results of this study suggest that the reactions involving CCH do not consistently result in efficient molecular growth, as pointed out for methanol.^63^ Consequently, in TMC-1, the H_2_CS + CCH reaction should be regarded as a destruction process and as a source of the HCS radical and HCCH rather than as a formation route for the C_3_H_2_S isomers. Therefore, the observed abundances of thiopropynal, cyclopropenethione, and propadienthione are likely controlled by alternative formation mechanisms, including other reactions summarized in Figure 1, or by additional pathways that remain to be proposed and/or investigated, such as the CH_3_ + CCS reaction.

## Conclusions

In this work, the gas-phase reaction between H_2_CS and CCH has been investigated through an accurate characterization of its reactive potential energy surface and subsequent kinetic simulations. The same methodology has been applied to the oxygen analogue H_2_CO + CCH reaction, for which experimental kinetic data are available. The systematic comparison between the two systems allowed us to point out similarities and differences.

Both reactions are dominated by a barrierless H-abstraction pathway leading to the formation of HCS/HCO together with acetylene, further confirming that H-abstraction is often the main channel for reactions involving the CCH radical.^63^ Although the H_2_CS + CCH potential energy surface presents more submerged pathways than its oxygen counterpart, these channels play only a minor role under interstellar conditions because the direct H-abstraction pathway is kinetically highly favored. In this regard, a comment is warranted on a recent paper that appeared in the arXiv^64^ while this article was in preparation. The arXiv manuscript reports the investigation of the H_2_CS + CCH and H_2_CS + CN reactions. The reactive potential energy surfaces there derived provide only a partial and limited picture compared to ours (this work for CCH and ref 14 for CN). Indeed, in ref 64, the authors only investigated addition products, without including any path for the H-abstraction channel, which is the predominant one in the H_2_CS + CCH system. However, by comparing our reactive PESs with theirs, also the addition pathways appear not to be exhaustively addressed in ref 64.

Overall, the present work provides the first detailed and accurate characterization of the H_2_CS + CCH reaction together with reliable rate coefficients that can be employed in astrochemical databases. Based on the theory-experiment comparison carried out for the H_2_CO + CCH → HCO + HCCH reaction, the reported rate coefficients are expected to have an accuracy of 15–20%.

## Supplementary Material



## Notes

Extended discussion on the derivation of the PST parameters employed in the modeling of the title reactions; table reporting the revDSD/junTZ and junChS relative energies for the H_2_CO + CCH reaction; table reporting the comparison between computed and experimental vibrational frequencies for H_2_CO, H_2_CS, HCO, HCS, HCCH, and CCH; pressure dependence of the H_2_CS + CCH and H_2_CO + CCH global rate coefficients (PDF)
