# Supplementary material for: Computational Study of the H2CS and H2CO Reactions with CCH: Mechanisms and Kinetic Implications for Interstellar Chemistry
Source: J Phys Chem A. 2026 Oct 12;130(44):8772–81. doi: 10.1021/acs.jpca.6c04860 (PMC13746815; doi:10.1021/acs.jpca.6c04860)
Supplement: Supplementary file 1 [file jp6c04860_si_001.pdf]

Supporting information for "A Computational Study of the H_2CS and H_2CO Reactions with CCH: Mechanisms and Kinetic Implications for Interstellar Chemistry"

Silvia Alessandrini,[*,†] Luigi Crisci,[†] and Cristina Puzzarini[*,‡]

† *Scuola Superiore Meridionale, Largo San Marcellino 10, 80138 Naples, Italy*

‡*Dipartimento di Chimica "Giacomo Ciamician", Alma Mater Studiorum - University of Bologna, Via P. Gobetti 85, Bologna, I-40129, Italy*

E-mail: silvia.alessandrini7@unibo.it; cristina.puzzarini@unibo.it

Long range coefficients for the PST modeling

Single-point energies were computed using the jun-ChS-F12 composite scheme (see main manuscript for details). For each channel ∼14 points were selected from Gaussian rigid-scan trajectories following the protocol of ref. 1: five core points at normalized coordinates $X \in \{0.0, 0.2, 0.4, 0.6, 1.0\}$ on $[R_{\min}, R_{\max}]$ ($R_{\min} = 1.7 \sum r_{\mathrm{cov}}$, $R_{\max} = 2 \sum r_{\mathrm{vdW}}$) plus ∼9 tail points at $R > R_{\max}$. When $R_{\min}$ is below the scan grid, the nearest point is substituted and two X-targets may collapse to the same grid point (reducing the core count to 4).

For H_2CS + CCH, the six innermost rigid-scan points converged to an incorrect electronic state; they were recomputed by seeding each point from the converged UHF orbitals of the adjacent outer point, with state consistency verified via $\langle \hat{S}^2 \rangle$ and $|\boldsymbol{\mu}|$ across all three UHF blocks.

Long-range potentials are fitted to

$$V_n(R) = -C_n/R^n, \qquad n = 4, 5, 6, \tag{1}$$

with $V(\infty) = 0$ (energies referenced to the largest-R point). Two fits are shown for each scan: 14-point (full dataset) and core-only (paper scheme).

HCO + CCH (MIN6, C···C)

Scan parameters

Reaction: HCO + CCH. Scan coordinate: R = C (HCO)···C (CCH) distance. Paper limits: $R_{\min} = 2.482$ Å, $R_{\max} = 6.800$ Å. Core points (†): 5 distinct grid points nearest to $X \in \{0.0, 0.2, 0.4, 0.6, 1.0\}$ on $[R_{\min}, R_{\max}]$.

Table S1: jun-ChS-F12 energies for the HCO + CCH (MIN6, C$\cdots$C) scan. $\Delta E = E(R) - E(R_{\max})$, referenced to $E_{\text{ref}} = -191.1644958100\,\mathbf{E}_{\text{h}}$ at $R = 10.2079$ Å. Core points (†) are those used in the paper fit scheme.

R (Å)	ΔE (kJ mol^{-1})	Core
3.2079	−6.6784	†
3.3079	−6.5311	†
4.2079	−3.0100	†
5.1079	−1.2150	†
6.8079	−0.2745	†
7.2079	−0.2000	
7.6079	−0.1460	
7.9079	−0.1149	
8.3079	−0.0821	
8.7079	−0.0568	
9.1079	−0.0368	
9.4079	−0.0245	
9.8079	−0.0110	
10.2079	0.0000	

Table S2: Long-range coefficients C_n and RMSD for $V_n(R) = -C_n/R^n$, HCO + HCCH (MIN6, C$\cdots$C) scan. "14-point": all available points. "5-point": core points only (paper scheme).

	14-point fit		5-point fit	
n	C_n (Å^n kJ mol^{-1})	RMSD (kJ mol^{-1})	C_n (Å^n kJ mol^{-1})	RMSD (kJ mol^{-1})
4	752.23	0.2209	753.01	0.3558
5	2471.32	0.3788	2470.88	0.6322
6	8031.27	0.5173	8029.76	0.8605

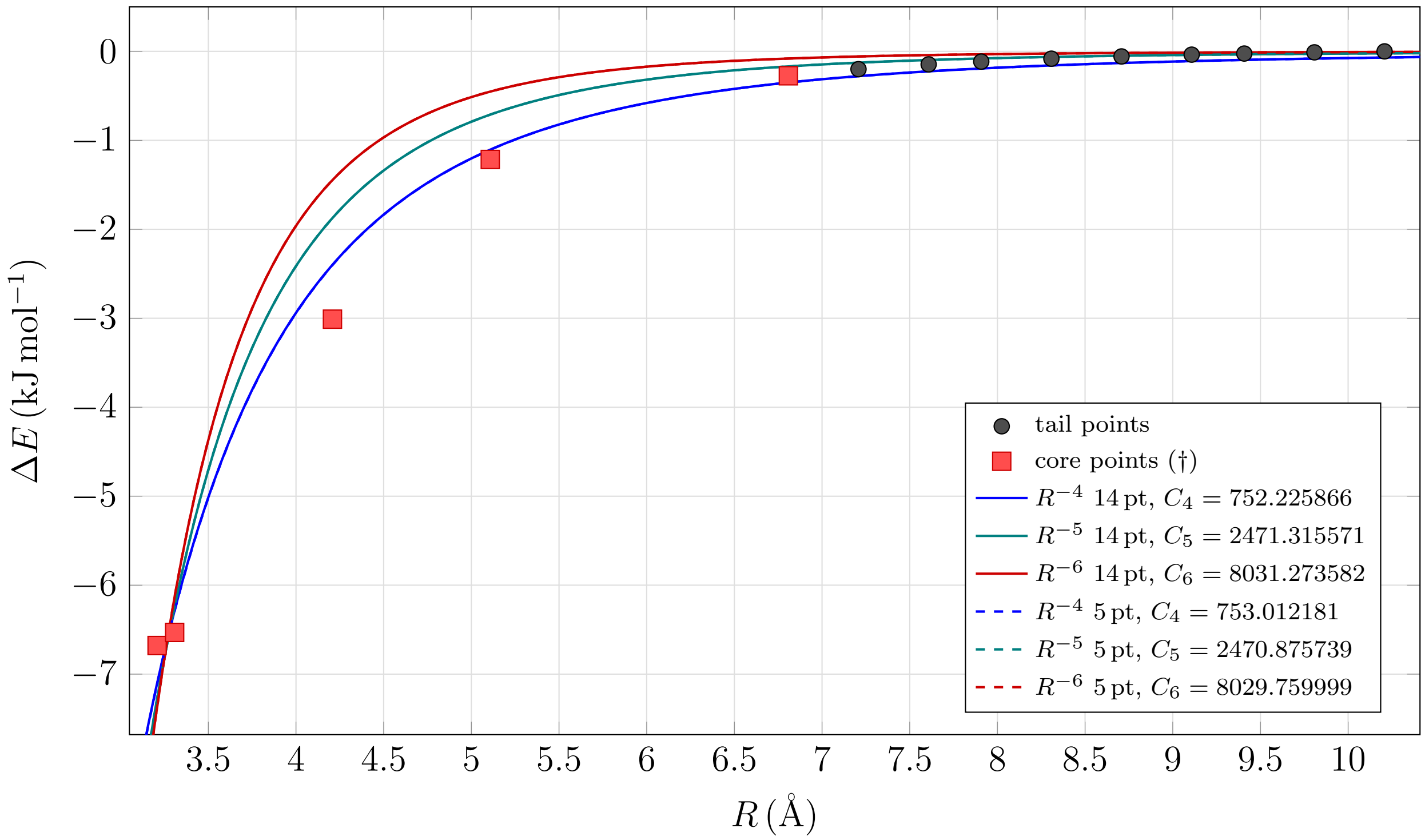

Figure S1: Long-range potential fits for the HCO + CCH (MIN6, C$\cdots$C) scan. Solid lines: fit to all 14 points. Dashed lines: fit to the 5 core points (paper scheme). $V_n(R) = -C_n/R^n$ with $V(\infty) = 0$.

H_2CO + CCH (PRC1, O$\cdots$C)

Scan parameters.

Reaction: H_2CO + CCH. Scan coordinate: R = O (H_2CO)$\cdots$C (CCH) distance. Paper limits: $R_{\min} = 2.363$ Å, $R_{\max} = 6.440$ Å. Core points (†): 5 distinct grid points nearest to $X \in \{0.0, 0.2, 0.4, 0.6, 1.0\}$ on $[R_{\min}, R_{\max}]$.

Table S3: jun-ChS-F12 energies for the H_2CO + CCH (PRC1, O$\cdots$C) scan. $\Delta E = E(R) - E(R_{\mathrm{max}})$, referenced to $E_{\mathrm{ref}} = -191.0911961600\,\mathbf{E}_{\mathrm{h}}$ at $R = 10.2411$ Å. Core points (†) are those used in the paper fit scheme.

R (Å)	ΔE (kJ mol^{-1})	Core
2.3411	−13.1446	†
3.1411	−6.9968	†
4.0411	−2.3429	†
4.8411	−0.9122	†
6.4411	−0.1787	†
6.8411	−0.1211	
7.2411	−0.0818	
7.7411	−0.0499	
8.1411	−0.0332	
8.5411	−0.0215	
8.9411	−0.0132	
9.4411	−0.0061	
9.8411	−0.0025	
10.2411	0.0000	

Table S4: Long-range coefficients C_n and RMSD for $V_n(R) = -C_n/R^n$, H_2CO + CCH (PRC1, O$\cdots$C) scan. "14-point": all available points. "5-point": core points only (paper scheme).

	14-point fit		5-point fit	
n	C_n (Å^n kJ mol^{-1})	RMSD (kJ mol^{-1})	C_n (Å^n kJ mol^{-1})	RMSD (kJ mol^{-1})
4	422.14	0.7809	422.24	1.3043
5	992.55	1.1127	992.53	1.8617
6	2306.45	1.3557	2306.40	2.2678

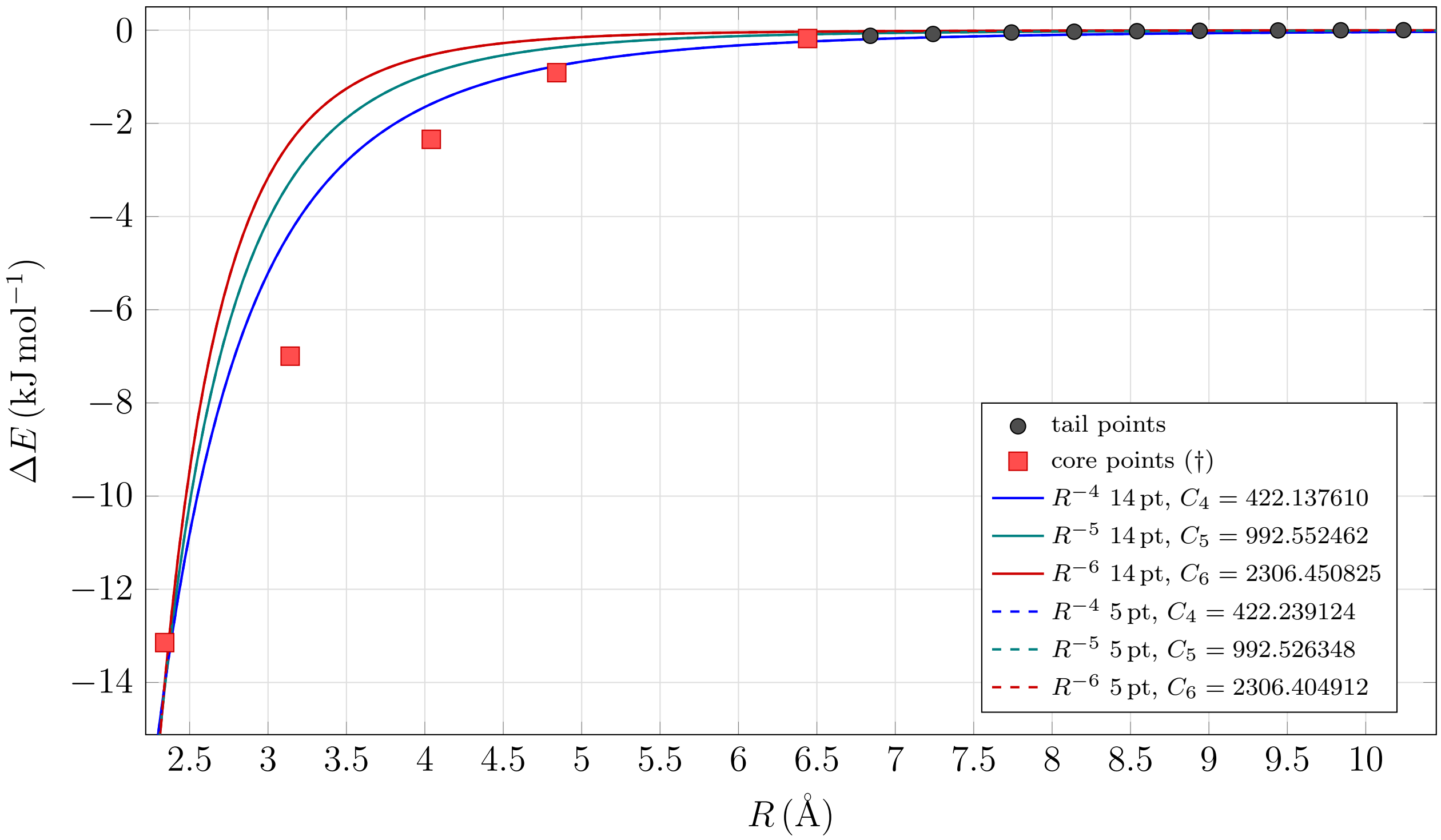

Figure S2: Long-range potential fits for the HCO + CCH (PRC1, O· · · C) scan. Solid lines: fit to all 14 points. Dashed lines: fit to the 5 core points (paper scheme). $V_n(R) = -C_n/R^n$ with $V(\infty) = 0$.

H_2CS + CCH (C· · · C, entrance channel)

Scan parameters.

Reaction: H_2CS + CCH. Scan coordinate: R = C (H_2CS)· · · C (CCH) distance. Paper limits: $R_{\min} = 2.482$ Å, $R_{\max} = 6.800$ Å. Core points (†): 4 distinct grid points nearest to $X \in \{0.0, 0.2, 0.4, 0.6, 1.0\}$ on $[R_{\min}, R_{\max}]$. Methods: revDSD-PBEP86-D3(BJ) (used for the potential) and jun-ChS-F12 (restricted-open, benchmark). Both scans share the same geometries.

Table S5: revDSD and jun-ChS-F12 energies for the H_2CS + CCH ($C\cdots C$) scan. $\Delta E = E(R) - E(R_{\mathrm{max}})$, each referenced to its own value at $R = 13.8979$ Å ($E_{\mathrm{ref}}^{\mathrm{revDSD}} = -513.5816825738\,\mathbf{E}_{\mathrm{h}}$, $E_{\mathrm{ref}}^{\mathrm{jun\text{-}ChS}} = -513.9003036500\,\mathbf{E}_{\mathrm{h}}$). Core points (†) are those used in the paper fit scheme.

R (Å)	$\Delta E_{\mathrm{revDSD}}$ (kJ mol^{-1})	$\Delta E_{\mathrm{jun\text{-}ChS}}$ (kJ mol^{-1})	Core
3.8979	−1.8925	−9.1255	†
4.1979	−1.3433	−7.6179	†
5.0979	−0.4291	−3.7709	†
6.7979	−0.0949	−1.2555	†
6.8979	−0.0894	−1.1819	
7.6979	−0.0589	−0.7540	
8.4979	−0.0402	−0.5059	
9.1979	−0.0289	−0.3612	
9.9979	−0.0199	−0.2451	
10.7979	−0.0135	−0.1630	
11.5979	−0.0088	−0.1024	
12.2979	−0.0056	−0.0630	
13.0979	−0.0025	−0.0277	
13.8979	0.0000	0.0000	

Table S6: Long-range coefficients C_n and RMSD for $V_n(R) = -C_n/R^n$, H_2CS + CCH ($C\cdots C$) scan (14-point fit, all available points). The revDSD scan is reproduced by the single-power form with a smaller RMSD than jun-ChS-F12.

	revDSD		jun-ChS-F12	
n	C_n (Å^n kJ mol^{-1})	RMSD (kJ mol^{-1})	C_n (Å^n kJ mol^{-1})	RMSD (kJ mol^{-1})
4	416.14	0.0730	2230.77	0.2407
5	1706.29	0.0225	9010.89	0.5173
6	6870.48	0.0357	35873.29	0.7327

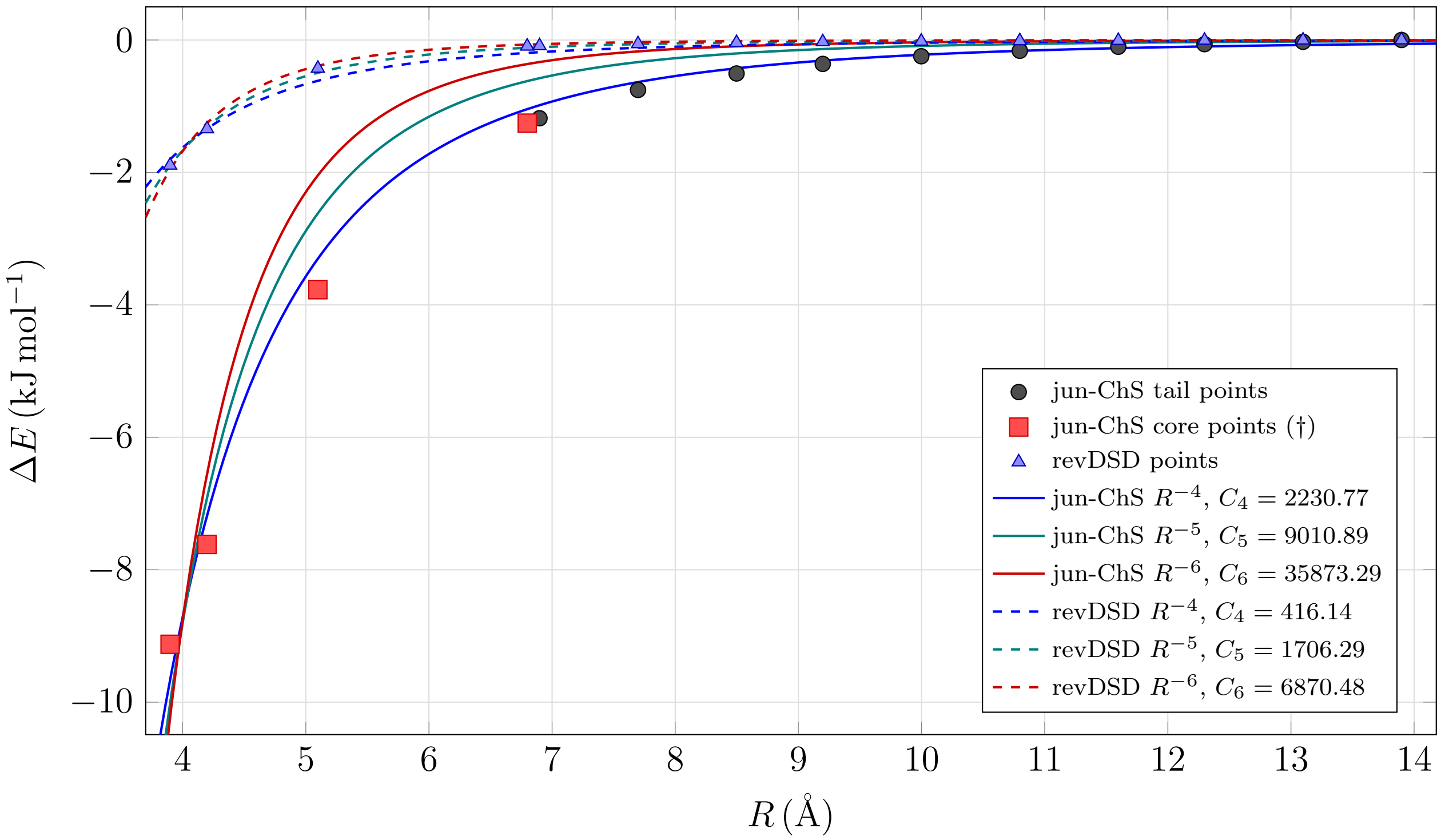

Figure S3: Long-range potential fits for the H_2CS + CCH (C$\cdots$C) scan. Solid lines: jun-ChS-F12 fits. Dashed lines: revDSD fits. $V_n(R) = -C_n/R^n$ with $V(\infty) = 0$ (14-point fit).

HCS + HCCH (exit channel, H$\cdots$C)

Scan parameters.

Reaction: HCS + HCCH. Scan coordinate: R = H (HCCH)$\cdots$C (HCS) distance. Paper limits: $R_{\min} = 1.768$ Å, $R_{\max} = 5.600$ Å. Core points (†): 4 distinct grid points nearest to $X \in \{0.0, 0.2, 0.4, 0.6, 1.0\}$ on $[R_{\min}, R_{\max}]$.

Table S7: jun-ChS-F12 energies for the HCS + HCCH (exit channel, H$\cdots$C) scan. $\Delta E = E(R) - E(R_{\text{max}})$, referenced to $E_{\text{ref}} = -513.9945290300\,\mathbf{E}_\text{h}$ at $R = 12.5652$ Å. Core points (†) are those used in the paper fit scheme.

R (Å)	ΔE (kJ mol^{-1})	Core
2.5652	−7.2606	†
3.2652	−4.7881	†
4.0652	−2.2927	†
5.5652	−0.6901	†
5.6652	−0.6437	
6.4652	−0.3800	
7.1652	−0.2513	
7.9652	−0.1604	
8.7652	−0.1022	
9.4652	−0.0679	
10.2652	−0.0407	
11.0652	−0.0220	
11.7652	−0.0101	
12.5652	0.0000	

Table S8: Long-range coefficients C_n and RMSD for $V_n(R) = -C_n/R^n$, HCS + HCCH (exit channel, H$\cdots$C) scan. "14-point": all available points. "4-point": core points only (paper scheme).

	14-point fit		4-point fit	
n	C_n (Å^n kJ mol^{-1})	RMSD (kJ mol^{-1})	C_n (Å^n kJ mol^{-1})	RMSD (kJ mol^{-1})
4	350.87	0.5899	350.12	1.0869
5	903.84	0.8090	902.40	1.4801
6	2298.09	0.9678	2296.19	1.7725

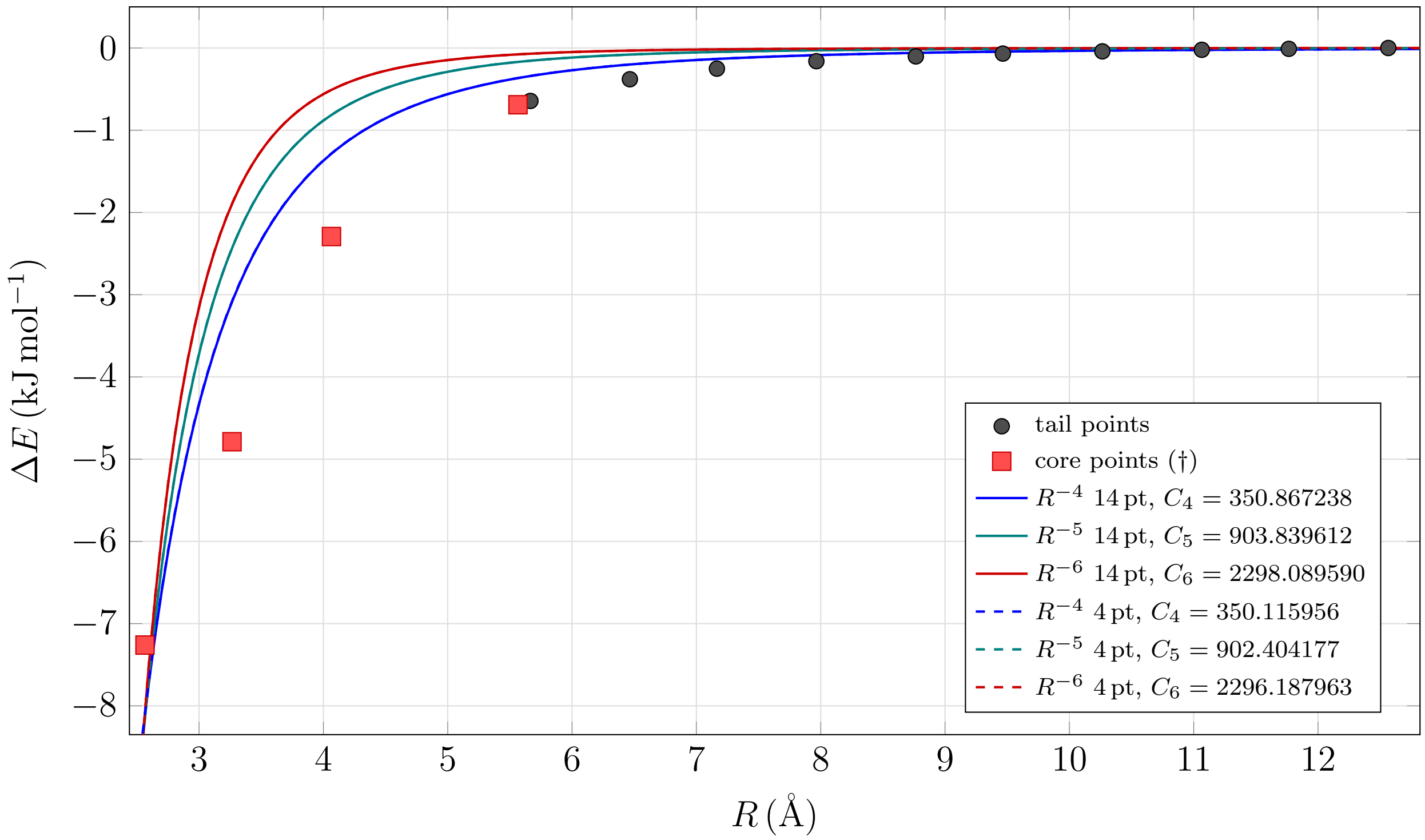

Figure S4: Long-range potential fits for the HCS + HCCH (exit channel, H···C) scan. Solid lines: fit to all 14 points. Dashed lines: fit to the 4 core points (paper scheme). $V_n(R) = -C_n/R^n$ with $V(\infty) = 0$.

Summary of long-range coefficients

Table S9: jun-ChS-F12 long-range coefficients C_n for all scans. $V_n(R) = -C_n/R^n$ (kJ mol^{-1}, R in Å). 14-point: all selected points. Core: paper scheme core points only. RMSD in kJ mol^{-1}.

		14-point fit		Core-only fit	
Scan	n	C_n (Å^n kJ mol^{-1})	RMSD	C_n (Å^n kJ mol^{-1})	RMSD
HCO + CCH (MIN6, C···C)	4	752.23	0.2209	753.01	0.3558
HCO + CCH (MIN6, C···C)	5	2471.32	0.3788	2470.88	0.6322
HCO + CCH (MIN6, C···C)	6	8031.27	0.5173	8029.76	0.8605
HCO + CCH (PRC1, O···C)	4	422.14	0.7809	422.24	1.3043
HCO + CCH (PRC1, O···C)	5	992.55	1.1127	992.53	1.8617
HCO + CCH (PRC1, O···C)	6	2306.45	1.3557	2306.40	2.2678
H_2CS + CCH (C···C, orbital following)	4	2230.77	0.2407	2226.37	0.4304
H_2CS + CCH (C···C, orbital following)	5	9010.89	0.5173	8975.78	0.8689
H_2CS + CCH (C···C, orbital following)	6	35873.29	0.7327	35766.38	1.2371
HCS + HCCH (exit channel, H···C)	4	350.87	0.5899	350.12	1.0869
HCS + HCCH (exit channel, H···C)	5	903.84	0.8090	902.40	1.4801
HCS + HCCH (exit channel, H···C)	6	2298.09	0.9678	2296.19	1.7725

The $H_2CO + CCH$ PES: energies

Table S10: Relative energies (kJ·mol^{-1})[a] of the stationary points of the H_2CO + CCH reactive PES. For labeling, see Figure 3 of the manuscript.

	revDSD/ junTZ[b]	junChS[b]		revDSD/[b] junTZ[b]	junChS[b]		revDSD/[b] junTZ[b]	junChS[b]
PRC1	-9.6	-5.9	TS2	-261.9	-252.5	TS22	-68.6	-59.7
MIN1	-199.0	-177.4	TS3	-80.6	-71.3	TS23	-189.7	-168.6
MIN2	-276.1	-266.6	TS4	-83.8	-73.9	TS24	-52.2	-31.4
MIN3	-283.7	-274.0	TS8	-100.6	-79.9	TS25	-159.2	-144.4
MIN4	-282.9	-267.7	TS7	-100.7	-88.5	TS26	24.5	35.0
MIN5	-293.0	-282.7	TS6	-103.3	-93.0	TS27	-18.4	-12.1
MIN6	-35.8	-23.5	TS5	-93.2	-81.2	TS28	-46.6	-22.6
MIN7	-174.9	-156.4	TS9	-161.5	-151.8	TS29	20.8	30.05
MIN8	-182.9	-172.8	TS10	47.3	62.3	TS30	-126.8	-111.8
MIN9	-155.6	-135.6	TS11	-96.1	-80.0	TS31	-64.9	-58.0
MIN10	-165.7	-145.8	TS12	-3.4	-2.1	TS32	-189.6	-165.7
MIN11	-86.4	-68.2	TS14	-120.8	-116.1	Pr1	-133.5	-110.8
MIN12	-381.8	-360.4	TS15	-87.7	-76.3	Pr2	-214.1	-192.8
MIN13	-131.1	-119.7	TS16	5.4	4.8	Pr3	-140.7	-113.4
MIN14	-252.0	-238.6	TS17	-83.42	-65.3	Pr4	-109.4	-82.6
MIN16	-213.4	-196.4	TS18	-179.9	-158.7			
MIN17	-134.0	-163.3	TS19	79.9	98.5			
MIN18	-339.6	-315.6	TS20	-25.5	-40.5			
TS1	-87.4	-73.8	TS21	-59.1	-51.3			

[a] The ZPE-corrected absolute energy (in Hartree, E_h) at the revDSD/junTZ level of H_2CO is -114.32877 E_h, while is -114.46873 E_h at the junChS level. For the absolute energy of CCH, see Table 1 of the manuscript.
[b] Harmonic ZPE corrections calculated at the revDSD/junTZ level of theory.

Vibrational frequencies of the reactants and products

Table S11: Comparison between best experimental (Exp.) and theoretical (Theo.) vibrational frequencies for H_2CS, H_2CO, CCH, HCCH, HCS, and HCO. "Err" stands for relative error expressed in %.

Mode	H_2CS			Mode	H_2CO		
	Exp.[2]	Theo.	Err. (%)		Exp.[2]	Theo.	Err. (%)
ν_1	3024.6	3177.3	5.1	ν_1	2843.3	3003.2	5.6
ν_2	2971.0	3088.4	3.9	ν_2	2782.5	2936.4	5.5
ν_3	1457.3	1503.1	3.1	ν_3	1746.0	1783.4	2.1
ν_4	1059.2	1089.5	2.9	ν_4	1500.2	1538.4	2.6
ν_5	991.00	1023.6	3.3	ν_5	1249.1	1268.5	1.6
ν_6	990.2	1008.6	1.9	ν_6	1167.3	1202.5	3.0
Mode	CCH			Mode	HCCH		
	Exp.[3]	Theo.	Err. (%)		Exp.[4]	Theo.	Err. (%)
ν_1	3299	3484.3	5.6	ν_1	3374(6)	3512.2	4.1
ν_2	1841	2281.6	23.9	ν_2	3289(3)	3419.1	4.0
ν_3	371.6	569.1	53.2	ν_3	1974(6)	2012.6	2.0
ν_4	371.6	569.1	53.2	ν_4	730(1)	758.1	3.8
				ν_5	730(1)	758.1	3.8
				ν_6	612(6)	619.2	1.2
				ν_7	612(6)	619.2	1.2
Mode	HCS			Mode	HCO		
	Best Theo.,[5a]	Theo.	Err. (%)		Exp. (Ar matrix)[6]	Theo.	Err. (%)
ν_1	3139.3	3136.6	0.09	ν_1	2483.2	2722.7	9.6
ν_2	1206.5	1349.5	11.8	ν_2	1863.5	1898.1	1.9
ν_3	826.5	814.3	1.5	ν_3	1085.5	1118.5	3.0

[a] Experimental values not available. Best Computed data from ref.[5] at the CCSD(T)/(CBS+CV)+ΔSR level of theory.

Pressure dependence

The collisional parameters for the H_2CO + CCH reaction were taken from ref. 7, while for H_2CS + CCH, a σ of 5.2 Å was estimated for the reactants's complex, while the ϵ was set to 500 K.

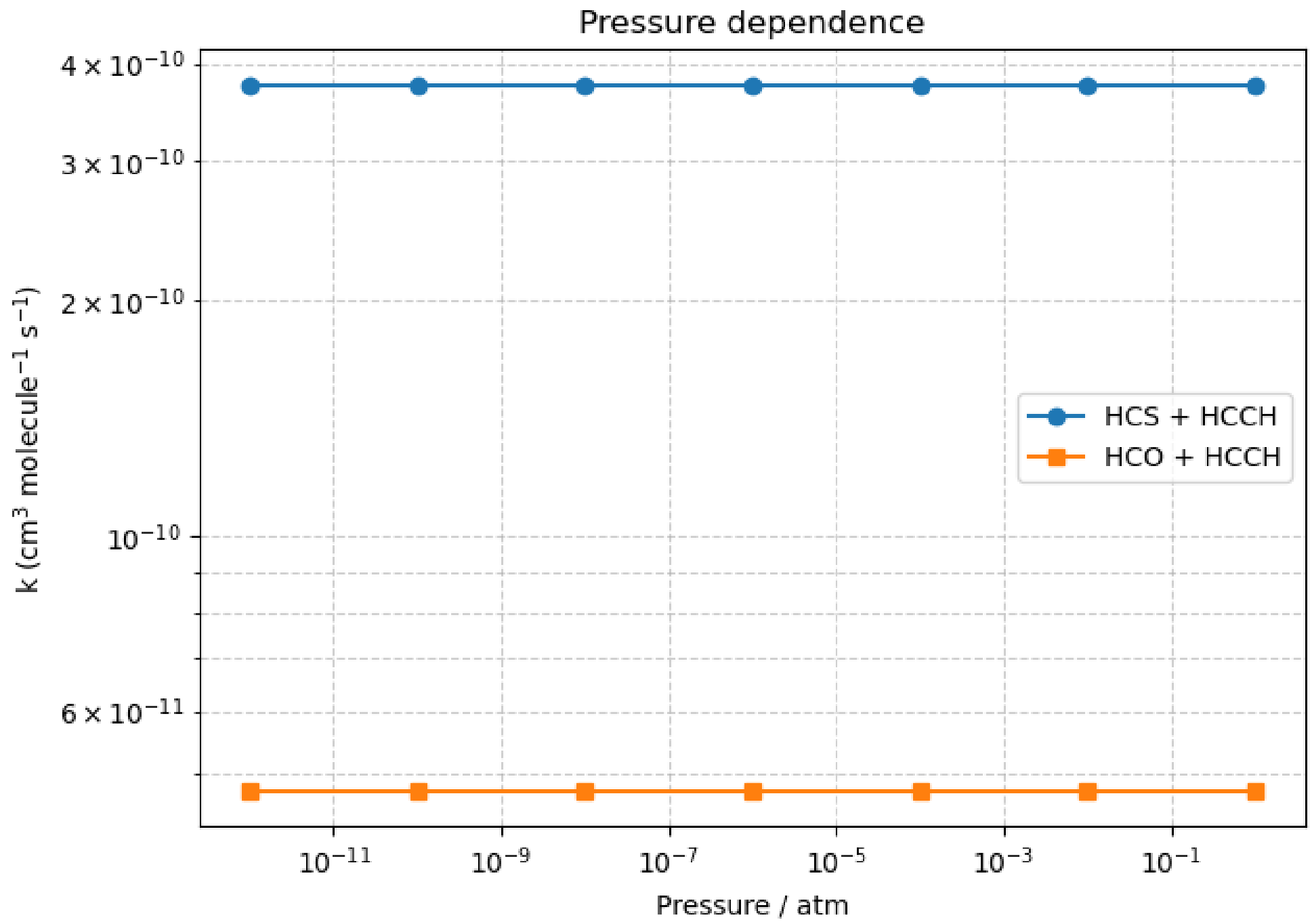

Figure S5: Pressure dependence at t=100 K of the global rate constants for the H_2CS + CCH → Pr2 and H_2CO + CCH → Pr2 reactions.

References

(1) Crisci, L.; Ballotta, B.; Mendolicchio, M.; Barone, V. Reduced Cost Computation and Exploitation of Accurate Radial Interaction Potentials for Barrier-Less Processes. *J. Chem. Theory Comput.* **2024**, *20*, 9435–9445.

(2) Clouthier, D.; Ramsay, D. The spectroscopy of formaldehyde and thioformaldehyde. *Annual Review of Physical Chemistry* **1983**, *34*, 31–58.

(3) Data, C. P. T. NIST Chemistry WebBook, NIST Standard Reference Database Number 69, 2005.

(4) Shimanouchi, T.; others *Tables of molecular vibrational frequencies*; US Government Printing Office Washington, DC, 1973.

(5) Puzzarini, C. The HCS/HSC and HCS^+/HSC^+ systems: molecular properties, isomerization, and energetics. *J. Chem. Phys.* **2005**, *123*.

(6) Ryazantsev, S. V.; Tyurin, D. A.; Feldman, V. I. Experimental determination of the absolute infrared absorption intensities of formyl radical HCO. *Spectrochim. Acta A Mol. Biomol. Spectrosc* **2017**, *187*, 39–42.

(7) West, N. A.; Li, L. H. D.; Millar, T. J.; Van de Sande, M.; Rutter, E.; Blitz, M. A.; Lehman, J. H.; Decin, L.; Heard, D. E. Experimental and Theoretical Study of the Low-temperature Kinetics of the Reaction of CN with CH_2O and Implications for Interstellar Environments. *Phys. Chem Chem. Phys.* **2023**, *25*, 7719–7733.
